# *In-Vitro* Studies on Selected Jordanian Plants as Dipeptidyl Peptidase-IV Inhibitors for Management of Diabetes Mellitus

**DOI:** 10.22037/ijpr.2020.1101232

**Published:** 2020

**Authors:** Nuha Sewidan, Reema Abu Khalaf, Hani Mohammad, Wa’ed Hammad

**Affiliations:** a *Department of Chemistry, Faculty of Arts and Sciences, University of Petra, Amman, Jordan. *; b *Department of Pharmacy, Faculty of Pharmacy, Al-Zaytoonah University of Jordan, Amman, Jordan.*

**Keywords:** Artemisia herba-alba, Calotropis procera, Diabetes mellitus, Dipeptidyl peptidase-IV, Ephedra foeminea, Hylocereus undatus, Marrubium vulgare

## Abstract

Diabetes mellitus is a chronic disease characterized by hyperglycemia mainly because of the absolute or relative deficiency of insulin hormone. The dipeptidyl peptidase-IV inhibitors represent a class of glucose-lowering agents potentiating the action of the incretin hormones glucagon-likepeptide-1 and glucose dependent insulinotropic polypeptide, which are secreted from the intestinal endocrine cells in response to food ingestion to stimulate insulin secretion from pancreatic beta cells. Natural products have been traditionally used for curing many diseases. In this study, *in-vitro* biological evaluation of the isolated compounds calotoxin, calotropin, pectolinarigenin, apigenin7-O-(3",6"-di-O-E-p-coumaroyl)-β-glycoside and extracts of *Calotropis procera*, *Ephedra foeminea*, *Artemisia herba-alba*, *Hylocereus undatus* and *Marrubium vulgare* showed potential inhibitory activity, where the butanol extract of *Calotropis procera* was found to have 85.3% inhibition of dipeptidyl peptidase-IV at 0.2 mg/100 µL concentration.

## Introduction

DM is a chronic progressive metabolic disorder characterized by hyperglycemia mainly because of the absolute or relative deficiency of insulin hormone (1, 2). According to the International Diabetes Federation reports, diabetes is a serious global health issue that continues to gain momentum, currently affecting 425 million people and set to affect over 690 million people by 2045 (3). 

Dietary habits and sedentary lifestyle are the major factors for rapidly rising incidence of DM among developing countries. Awareness about diabetes complications and consequent improvement in dietary knowledge, attitude, and practices lead to better control of the disease (4). The goals of therapy for diabetes are to alleviate the symptoms related to hyperglycemia and to prevent or reduce the complications of diabetes that can be acute as hypoglycemia, diabetic ketoacidosis and hyperosmolar coma, or chronic as microvascular and macrovascular alterations (5).

Dipeptidyl peptidase-IV (DPP-IV) inhibitors have been widely used as outstanding blood glucose-dependent antidiabetic agents for patients with type II DM (6-8). These inhibitors prevent the degradation of incretins, glucagon-likepeptide-1 (GLP-1) and glucose dependent insulinotropic polypeptide (GIP), by DPP-IV enzyme and therefore elevate their endogenous levels. GLP-1 stimulates insulin secretion from β-cells in a glucose-dependent manner, suppresses glucagon secretion from α-cells, and inhibits hepatic glucose production, eventually contributing to the antihyperglycemic effect. In addition, DPP-IV inhibitors preserve the β-cell mass (9, 10).

Natural products are characterized by their chemical diversity and being a good source of a range of bioactive structures including antidiabetic compounds. Rational drug design has been widely accomplished, to discover and optimize innovative leads for different molecular targets of type II diabetes including DPP-IV, α-glucosidase, peroxisome proliferator-activated receptor gamma (PPARγ), glycogen synthase kinase-3β (GSK-3β), glucokinase (GK), and others (9).

Natural products have been traditionally used for curing many diseases against various infectious agents such as bacteria, viruses, fungi, protozoans and worms; and are widely used for treatment of different diseases and physiological disorders (11). Numerous plants are traditionally used for the treatment of DM, but very little is known about the mechanism of action of antidiabetic activity of these medicinal plants. Therefore, the aim of this study was to evaluate the DPP-IV inhibitory potential of *Calotropis procera*,* Artemisia herba-alba*, *Ephedra foeminea*, *Hylocereus undatus*,* Marrubium vulgare *extracts, and additional four isolated compounds.

**Figure 1:**
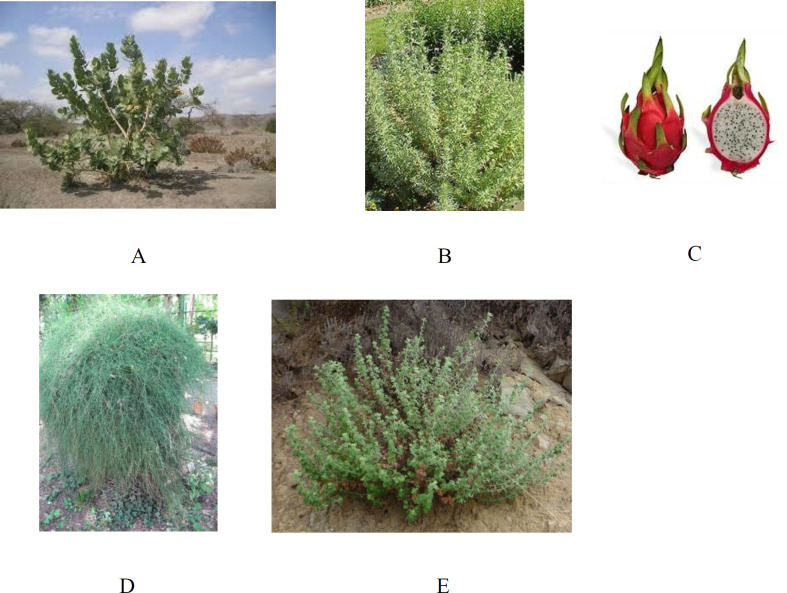
Showing the tested plants: (A)* Calotropis procera*, (B) *Artemisia herba-alba*, (C) *Hylocereus undatus* fruit, (D)* Ephedra foeminea*, and (E) *Marrubium vulgare*

*Calotropis procera* Linn, known historically as apple of Sodom and named by Edwaed Robinson, grows wild from West Africa to South East Asia and belongs to the *Asclepiadaceae* family (Figure 1). Most members of the family have milky juice, flowers with five united petals, pod like fruits, and usually tufted seeds. It includes more than 180 genera and about 2200 species of tropical herbs or shrubby climbers. Two species belonging to this family, *Calotropis procera* and *Calotropis gigantea*, are of economic importance. Active components of *Calotropis procera *(known in Arabic as Al oshar) displayed cytostatic, cytotoxic, wound healing, procoagulant, analgesic, anticonvulsant, antiarthritic, antidiabetic, hepatoprotective, antifertility, antipyretic, anticoccidial, anticancer, and anti-inflammatory properties. Sterols, triterpenes, flavonoids, and cardiac glycosides (cardenolides) have been isolated from *Calotropis procera* (11, 12)*.*

*Artemisia herba-alba* is thought to be the plant translated as “wormwood” in English-language versions of the Bible (*apsinthos* in the Greek text) (Figure 1). It is from the *Asteraceae* family. *Artemisia herba-alba* is a chamaeophyte that grows to 20–40 cm (8–16 in). Leaves are strongly aromatic and covered with fine glandular hairs that reflect sunlight giving a greyish aspect to the shrub. The leaves of sterile shoots are grey, petiolate, ovate to orbicular in outline; whereas, the leaves of flowering stems, more abundant in winter, are much smaller. The plants flower from September to December. *Artemisia herba-alba*, grows commonly on the dry steppes of the Mediterranean regions, in Northern Africa, Western Asia and Southwestern Europe also known as desert wormwood (known in Arabic as shih), has been traditionally used in the treatment of a variety of ailments such as cold, diabetes, and bronchitis (13, 14). Herbal tea from this species has been used as analgesic, antibacterial, antispasmodic, and hemostatic agents (15). During an ethnopharmacological survey carried out among the Bedouins of the Negev desert, it was found that *Artemisia herba-alba* relieved stomach disorders (16). Various secondary metabolites have been isolated from *Artemisia herba-alba,* including sesquiterpene lactones that are considered the most important and occur with great structural diversity. Additional studies have focused on flavonoids and essential oils (13).

Pitahaya or dragon fruit or Belle-of-the-night are the common names of *Hylocereus undatus* (*Pitaya blanca* or white-fleshed pitahaya) belonging to the *Cactaceae* family (Figure 1). It originates from tropical and subtropical America and belongs to a group of fruit trees considered promising for farming, which are distributed in Costa Rica, Venezuela, Panama, Uruguay, Brazil, Colombia, and Mexico (17). For its nutritional importance and culinary applications, pitaya can be utilized for hyperglycemia, as a diuretic, and a healing agent. The seeds have a laxative effect, the fruit has an effect on gastritis, and the stalk and flowers are also used for kidney problems. Extracts from dragon fruit have been associated as central nervous system stimulants and regulators of blood pressure, sleep, hunger and thirst. The fatty acid, phenolic, tocopherol, and sterol contents of the extracted plant were analysed (18).

*Ephedra* is likely one of the oldest medicinal plants still currently in use (Figure 1). *Ephedra* is a ubiquitous genus of gymnosperm shrubs that grow in temperate and subtropical regions usually on shores or in sandy soils under direct sunlight throughout North and Central America, Europe, Africa and Asia (19). In folk medicine, the extracts of *Ephedra foeminea *(*Ephedraceae*) named by Peter Forsskal are commonly used to treat cancer (20). It was used in the treatment and/or prophylaxis of various conditions such as asthma, nasal congestion, and hypotension caused by spinal anaesthesia and urinary incontinence Phytochemical investigations showed that *Ephedra foeminea *contains high contents of benzaldehyde, phenolics, and flavonoid compounds. Among all *Ephedra* species *Ephedra foeminea* lacks epherdrin alkaloid (21).

*Marrubium vulgare*, belonging to the *Lamiaceae* or *Labiatae* family, habitats in Europe, the Mediterranean, and Asia (Figure 1). Horehound which is the common name for *Marrubium vulgare* has been mentioned in conjunction with medicinal use dating at least back to the 1st century BC, where it appeared as a remedy for respiratory ailments in the treatise *De Medicina* by Roman encyclopaedist Aulus Cornelius Celsus. It is an aromatic herbaceous perennial plant used in traditional medicine in some countries such as Jordan in the treatment of diabetes and wounds. The plant is reported to possess vasorelaxant, antihypertensive, analgesic, anti-inflammatory, and antioxidant properties. On the other hand, some studies reported that the antioxidant effect of the plant extract is due to its flavonoid content. Earlier phytochemical investigations of *Marrubium vulgare *led to the characterization of the diterpene marrubiin which is the major constituent of the plant, and exhibits potent antinociceptive properties and vasorelaxant activity, in addition to marruboside and marrubic acid (22).

Several studies have been performed on the effect of various extracts of *Calotropis procera *(12) *Artemisia herba-alba *(23) and *Hylocereus undatus* (24) on Diabetes mellitus (DM) while no studies were reported on* Ephedra foeminea *extracts.

The research began after some local citizens having diabetes mellitus, claimed to be cured after drinking tea from *Marrubium vulgare* for several months. The tea was prepared by cooking the plant for 2 h in boiling hot water. Since four of the herbs under study, *Calotropis procera, Marrubium vulgare*, *Artemisia herba-alba, *and *Ephedra foeminea* grow wildly in Jordan it was a challenge to have a scientific study on these plants.

**Figure 2:**
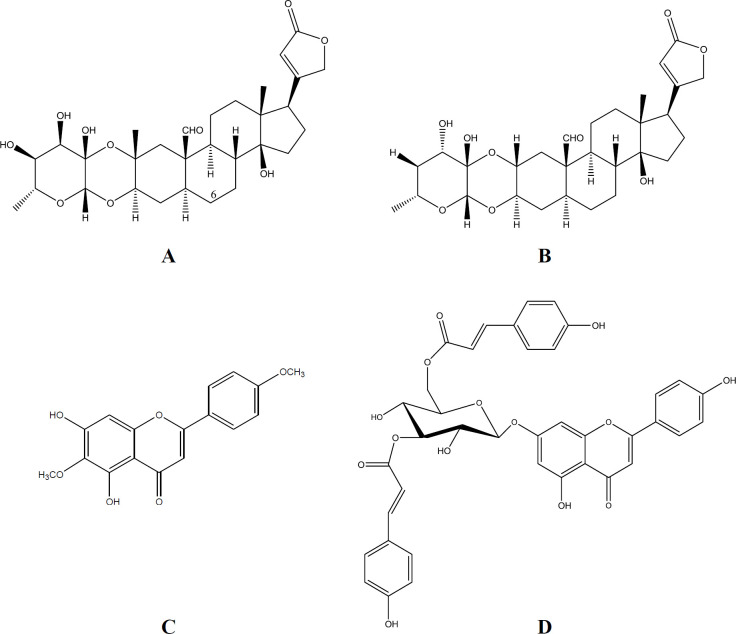
Chemical structures of (A**) **calotoxin,** (**B) calotropin,** (**C**) **pectolinarigenin,** (**D) apigenin7-O-(3",6"-di-O-E-p-coumaroyl)-β-glycoside

Calotropin and calotoxin (Figure 2), isolated and identified from *Calotropis procera *(25), belong to the cardenolide cardiac glycosides family, which has been recognized for its antidiabetic effect (26). Pectolinarigenin, a flavonoid, and apigenin7-O-(3”,6”-di-O-E-p-coumaroyl)-β-glycoside, a flavonoid glycoside, (Figure 2) have been isolated from *Marrubium vulgare *(Figure 1) (22). Flavonoids and glycosides represent a beneficial group of naturally occurring compounds with hypoglycemic potential (27).

## Experimental

*Chemicals and instruments*

*In-vitro* DPP-IV inhibitory activity of the isolated compounds and plant extracts were evaluated using a commercially available kit (DPP4 Inhibitor Screening Kit (Fluorometric, BioVision, USA).

AFLX800TBI Microplate Fluorimeter was used in the *in-vitro* bioassay (BioTek Instruments, Winooski, VT, USA) at the Faculty of Pharmacy, Al-Zaytoonah University of Jordan.

*In-vitro DPP-IV inhibitory assay*

The tested samples were initially dissolved in DMSO to yield stock solutions and subsequently diluted to the required concentrations using distilled water and then were added to the assay well in a final volume of 10 µL. The final concentration of DMSO was adjusted to 0.1%. The percentage of residual activity of DPP-IV was determined for each sample by comparing the activity of DPP-IV in the presence and absence of the tested sample. DPP-IV was not affected by DMSO. Negative controls lacking human recombinant DPP-IV were used as background. Sitagliptin was used as a positive control. All measurements were conducted in duplicates.

*Plants material and preparation of plant extract*

*Calotropis procera* and *Marrubium vulgare* were identified by prof. Barakat Abu Ermeleh from the College of Agriculture, The University of Jordan and deposited in its herbarium.* Ephedra foeminea and Artemisia herba-alba *were characterised by Dr Kenza Mansour from the College of Pharmacy and Medical studies, University of Petra.

*Calotropis procera*

The aerial part of *Calotropis procera* was collected from Dead Sea area, 30 Km south of Amman (Jordan) during the flowering period (April-May 2017). After collection, the plant was dried and ground (2 kg) then defatted by extraction with petroleum ether (2 L) at room temperature for 7 days. The residual plant material was then extracted with ethanol at room temperature (4 L, 7 days). The crude ethanolic extract was evaporated under reduced pressure and kept for further use. The resulting crude extract was partitioned between chloroform and water (1:1, 2 L). The chloroform layer was then evaporated under vacuum to give a gummy residue, which was afterwards partitioned between 10% aqueous methanol and hexane (1:1, 2 L). The lower layer contained aqueous methanol extract while the upper layer provided the hexane fraction. The water layer was extracted with *n*-butanol to provide the butanol fraction and the water extract.

*Latex from Calotropis procera*

When the aerial part of *Calotropis procera* was collected, fresh 20 g of its latex was carefully collected in small glass sample bottles. The latex was extracted 3 times (3x 25 mL) with methanol, evaporated under reduced pressure and kept at T = 4 °C for further use.

*Isolation of calotoxin and calotropin from Calotropis procera (* 25 *)*

The aqueous methanol fraction (150 g) was adsorbed on 150 g silica gel 60 and subjected to column chromatography (L × ID: 53 × 5.8 cm) using 500 g of the same adsorbent packed in chloroform. The polarity of the eluent was increased gradually with methanol until 100% methanol was used. A total of 100 fractions (500 mL) were collected and grouped into 8 groups according to their TLC behaviour. Further purification was done for each of these groups by a combination of column and thin layer chromatography. Fraction 4 was isolated as an impure solid and purified by washing with distilled methanol. It gives blue colour when sprayed with anisaldehyde followed by heating. This compound was identified using ^1^H and ^13^C NMR as well as mass spectroscopy as calotropin. Fraction 5 gave an impure solid, which was washed several times with acetonitrile to afford a white solid identified as calotoxin.

*Ephedra foeminea*

The plant was collected from West bank in summer 2017. Ten grams of the whole plant was ground and soaked in 250 mL of ethanol for one week. The ethanolic extract was then evaporated under reduced pressure and kept for further use.

*Artemisia herba-alba*

The plant was obtained from the local market in Jordan. Five grams of the whole plant was treated similarly to *Ephedra foeminea.*

*Hylocereus undatus*

Dragon fruits were obtained from local market in Jordan. All regents were obtained from Sigma-Aldrich. Two batches of 100 g of the bulb were homogenized in 100 mL ethanol and ethanol: water (1:1, v/v). The samples were centrifuged at T = 4 °C for 20 min, the clear extracts were obtained, dried under vacuum and kept in dark vessels. The peels of red dragon fruit were cut into pieces, dried and soaked in ethanol for 7 days. The ethanol extract was dried under vacuum and was saved.

*Isolation of pectolinarigenin and apigenin7-O-(3*”*,6*”*-di-O-E-p-coumaroyl)-β-glycoside from Marrubium vulgare (*22*)*

*Marrubium vulgare* was collected from Bader area to the west of Jordan during the flowering period (April-June 2017). The plant material was dried and ground to fine powder (18 kg) then was fractionated into different extracts like *Calotropis procera*. The methanol extract was loaded on a large column from which different groups were obtained.

Fraction 2 precipitated an impure solid which was recrystallized from methanol. A pure yellow solid was obtained and identified as pectolinarigenin. This flavonoid was isolated for the first time from *Marrubium* genera.

On TLC, fraction 6 showed two UV active spots. Purification of this fraction was performed on sephadex column eluted with (1: 1) CHCl_3_: MeOH as solvent system. Further purification was done on TLC plates using CHCl_3_: MeOH (8: 2) as an eluent. The compound with higher R_f_ value was identified as apigenin7-O-(3”,6”-di-O-*E*-*p*-coumaroyl)-β-glucoside, while the lower R_f_ value compound was identified as apigenin 7-(6”-*E*-*p*-coumaroyl) glucoside.

## Results and Discussion

The results of the *in-vitro* DPP-IV inhibition bioassay, presented in Table 1 demonstrate that all isolated compounds and plant extracts have an appreciable inhibitory activity against DPP-IV. The results were calculated using the following equation:

$\mathrm{Inhibition\%}=[1-\frac{\mathrm{nhibitor read}–\mathrm{Blank read}}{\mathrm{Positive control}-\mathrm{Negative control}}]\times 100$

As can be seen from the table, the tested ethanol extract of *Ephedra foeminea*, ethanol extract of *Hylocereus undatus* fruits, and butanol extract of *Calotropis procera *possess comparable inhibitory activity against DPP-IV enzyme to a known inhibitor, sitagliptin. The butanol extract of *Calotropis procera *showed the best DPP-IV inhibitory activity of 85.3% at a concentration of 0.2 mg/100 µL.

Flavonoids bioactive constituents have been reported to preserve β-cell integrity and function by scavenging free radicles in the system and therefore protect against the progression of DM (28). That encourages us to isolate the flavonoids: pectolinarigenin and apigenin7-O-(3”,6”-di-O-E-p-coumaroyl)-β-glycoside from *Marrubium vulgare *(22) followed by evaluation of their DPP-IV inhibitory activity (Figure 2). Apigenin7-O-(3”,6”-di-O-E-p-coumaroyl)-β-glycoside was found to have a promising antidiabetic activity with a DPP-IV inhibitory activity of 48.9% at 100 µM concentration.

It is evident from the results that the DPP-IV inhibitory activity of *Calotropis procera* extract is higher than that of any of its constituents, calotoxin, and calotropin (Figure 2). The potent inhibitory activity of the extract may be due to synergistic effect of the phytochemicals present in it. Moreover, it was found that ethanol extract of *Calotropis procera* leaf contains flavonoids, reducing sugar, and steroids (12).

The inhibitory activity of the tested extracts and isolated compounds toward DPP-IV suggest that active components of the extracts and the isolated compounds bind to the active site of the enzyme. This can disturb the binding of the enzyme to its normal substrate due to conformational changes, thus slowing the breaking down of GLP-1 and GIP. Consequently, the concentration of glucose in the blood is maintained thus controlling hyperglycemia and its complications.

**Table 1:** *In-vitro* bioactivities of the isolated compounds and plant extracts against DPP-IV Enzyme

**Name**	**Part used**	**Solvent used**	**Sample Concentration**	inhibition%*
*Calotropis procera*	Aerial	Ethanol	0.2 mg/100 µL	53.0 ± 0.69
*Calotropis procera*	Aerial	Butanol	0.2 mg/100 µL	85.3 ± 0.67
*Calotropis procera*	Aerial	Hexane	0.2 mg/100 µL	39.9 ± 0.90
*Calotropis procera*	Aerial	Petroleum Ether	0.2 mg/100 µL	31.9 ± 0.75
*Calotropis procera*	Latex	Methanol	0.2 mg/100 µL	14.0 ± 0.65
*Ephedra foeminea*	Whole	Ethanol	10 mg/100 µL	83.4 ± 0.78
*Hylocereus undatus*	Fruit	Ethanol & Water	10 mg/100 µL	26.8 ± 0.55
*Hylocereus undatus*	Peel	Ethanol	10 mg/100 µL	62.3 ± 0.63
*Hylocereus undatus*	Fruit	Ethanol	10 mg/100 µL	84.2 ± 0.72
*Artemisia * *herba-alba*	Whole	Ethanol	10 mg/100 µL	26.2 ± 0.61
Calotoxin	--	--	100 µM	31.0 ± 0.78
Calotropin	--	--	100 µM	19.9 ± 0.85
Pectolinarigenin	--	--	100 µM	34.7 ± 0.59
Apigenin7-O-(3",6"-di-O-E-p-coumaroyl)-β-glycoside	--	--	100 µM	48.9 ± 0.88
Sitagliptin	--	--	100 µM	88.3 ± 0.54

*Data is expressed as Mean ± SD, n = 2.

## Conclusion

This work indicated that the screened plants and isolated compounds have inhibitory activity against DPP-IV enzyme. Butanol extract of the aerial parts of *Calotropis procera* possess a significant potential as a source of lead compounds in the development of antidiabetic agents targeting DPP-IV.
